# Cuproptosis at the Crossroads of Tumor Microenvironment Inflammation and Metabolic Rewiring Mechanisms and Therapeutic Opportunities

**DOI:** 10.1155/mi/2952331

**Published:** 2026-03-09

**Authors:** Yan Wang, Yunfei Zhu, Yanxin Wang, Yutong Liu, Pengfei Wang, Mingkun Yu

**Affiliations:** ^1^ Graduate School, Heilongjiang University of Chinese Medicine, Harbin, China, hljucm.edu.cn; ^2^ Department of Oncology, Binzhou Medical College Affiliated Traditional Chinese Medicine Hospital, Binzhou, China, bzmc.edu.cn; ^3^ College of Traditional Chinese Medicine, Binzhou Medical University, Yantai, Shandong, China, bzmc.edu.cn; ^4^ Acupuncture and Massage College, Changchun University of Chinese Medicine, Changchun, China, ccucm.edu.cn; ^5^ Department of Endocrinology, Binzhou Medical College Affiliated Traditional Chinese Medicine Hospital, Binzhou, China, bzmc.edu.cn; ^6^ Binzhou Key Laboratory of Intelligent Creation and Functional Food Development of Medicine and Food Homologous Resources, Binzhou Traditional Chinese Medicine Hospital, Binzhou, China, bzmc.edu.cn

## Abstract

Metabolic reorganization and chronic inflammation are dynamic and diverse events in the tumor microenvironment (TME) that cause tumor propagation, spreading, and resistance to treatment. However, copper‐induced cell death (cuproptosis), which is a newly discovered form of regulated cell death (RCD) induced by high intracellular copper ions and is a common location coupled with the tricarboxylic acid cycle (TCA), has promptly become a hub of metal homeostasis, cellular metabolism, and immune regulation within the TME. This review will consider cuproptotic molecular pathways. We focus on how inflammatory cytokines and hypoxia directly influence copper uptake, intracellular traffic, and the cell death threshold. Compared with other more predominant types of RCD, we highlight the capacity of cuproptosis to trigger numerous potent immunogenic reactions by releasing damage‐associated molecular patterns (DAMPs), including calreticulin (CRT), ATP, HMGB1, and type I interferons (IFNs). The reciprocal crosstalk between copper dysregulation and chronic inflammation fuels tumor angiogenesis and invasion by fostering “copper addiction.” However, this metabolic dependency simultaneously creates a distinct vulnerability. We examine how inducing cuproptosis—conceptually distinct from simple copper accumulation—exploits this fragility to release DAMPs and reprogram the immune microenvironment of “cold” tumors. Such therapeutic strategies, spanning copper ionophores, chelators, and novel nanomedicines, offer significant potential to synergize with immunotherapies and overcome treatment resistance. Despite encouraging preclinical research results, some obstacles still need to be overcome, such as the cuproptosis‐specific modulation’s functionality, systemic toxicity reduction, and tumor heterogeneity. In order to transfer the curing power of cuproptosis directly into the medical practice, several key issues must be addressed in the future. These involve optimizing in vivo models to enhance physiological relevance, measuring cell‐type‐specific sensitivity, identifying reliable biomarkers, and utilizing highly sensitive modulators. The benefits of cuproptosis therapy will inevitably lead to the development of a novel healing strategy, which involves reorganizing the inflamed and immunosuppressive TME through cuproptosis, resulting in sustained and effective antitumor effects.

## 1. Introduction

The tumor microenvironment (TME) is called an ecosystem. It contains the cancer cells and the large number of nonmalignant cells, the immune cells, stromal cells, endothelial cells, and so on, that are constituting a dynamic and evolving extracellular matrix [1]. This perpetual restructuring of this microenvironment plays a critical role in the process of stimulating tumorigenesis, progression, metastasis, and tumor resistance to treatment [2]. The center of this structure is the specific inflammation that activates the formation of tumors continuously. It is so much a part and parcel of the cancer that it is considered one of the cancer hallmarks [3]. Chronic inflammatory diseases accumulate a self‐perpetuating, pro‐tumor environment with more molecules, such as IL‐1β and TNF‐α. These nourish proliferation and invasion/metastasis and promote transfer to other places [4]. In addition, such an inflammatory scene dramatically shapes the regional immune environment, promoting the accumulation and triggering of important immunosuppressive cells [5]. The most prominent include tumor‐associated macrophages (TAMs) and myeloid‐derived suppressor cells (MDSCs), which are actively involved in destroying antitumor surveillance through the production of suppressing cytokines, depletion of essential amino acids, and augmentation of immune checkpoints. Besides the consequences of TME inflammation to immune evasion, it induces angiogenesis to maintain the pace of metabolic needs of the tumor [6]. Also, it induces more reactive oxygen and nitrogen species in such a manner that the DNA of the tumor becomes unstable [7]. Together, these factors create a fertile ground that promotes cancer propagation and enhances metastasis.

The formation of cell death is a significant aspect that draws attention within this complex network of communications, as it is a key component in shaping the TME immunological environment and ultimately influencing treatment outcomes. Numerous forms of regulated cell death (RCD) have been characterized, such as apoptosis, necroptosis, pyroptosis, and ferroptosis. These pathways differ not only in their molecular machinery but also vary dramatically in their immunological effects [8]. Illustratively, apoptosis is, as a rule, immunologically silent; the immunosilence is primarily due to the cell’s sequential dissection, which can inhibit inflammation and even induce immune tolerance [9]. However, the inflammation response named immunogenic cell death (ICD) is activated by numerous other pathways of RCD, such as pyroptosis and necroptosis. It initiates an avalanche of damage‐associated molecular patterns (DAMPs), such as ATP, HMGB1, and type I interferon (IFNs), toward the surrounding spaces to attract and activate an immune response actively. They also act as internal adjuvants and strongly enhance antigen‐presenting cells, such as the dendritic cells (DCs), leading to effective and long‐lasting adaptive immune responses against tumor antigens [10]. However, there is so much heterogeneity, including intra and intertumor, and the extraordinary plasticity of the TME, that it is in no way sufficient to understand its complex regulatory circuits and heterogeneous immune responses, which implies that the point of view is still not sufficient to explain its complex and heterogeneous therapy responses fully. In turn, continuing research and the descriptive characterization of new RCD pathways are crucial to explaining the further dynamic development of the TME, identifying new targets to be addressed through treatment, and developing more effective anticancer strategies.

Most recently, a novel RCD pathway called copper death (cuproptosis) was discovered and officially named in 2022 at the leading edge of research in the field of oncology. Cuproptosis, a distinctly different mode of cell death, is activated upon excess copper ions inside the cells. The most momentous occurrence is the binding of these ions to the lipoylated protein constituents of the Tricarboxylic acid cycle (TCA cycle) in the mitochondria, which have high affinity and direct binding. This noncatalytic and destructive interaction causes irreversible aggregation and inactivation of key metabolic proteins. This strongly correlates with severe proteotoxic stress in the cells, triggering a cascade of downstream responses such as rapid degradation of iron–sulfur (Fe–S) cluster proteins and mitochondrial respiratory chain collapse, which leads to the death of the cells [11].

The main idea of the review is that cuproptosis is not only a never‐before‐described cell death pathway but also one of the more important and previously unknown intersections between core cellular metabolism (TCA cycle), metal homeostasis (copper regulation), and immune‐inflammatory ecology in the TME. To achieve this, we will begin by examining how the inflammatory TME regulates cellular copper uptake, efflux, and distribution through the release of cytokines, changes in local oxygen levels, and remodeling of cellular metabolism—thereby influencing the cuproptosis induction threshold. Second, we will discuss how cuproptosis serves as an effective signal that alters the immune cell composition and overall inflammatory state of the TME due to the release of certain DAMPs. Exploring such a complex interaction, one can make an effort to justify the possibility of targeting the cuproptosis pathway. This pathway is fragile, and any alteration in nature can cut the damaging linkage between chronic inflammation and cancer growth. Finally, the review appears to support both the development of next‐generation cancer therapeutics, and the exploration of new therapeutic targets.

## 2. Molecular Mechanisms and Regulation of Cuproptosis

In contrast to an isolated cellular event, cuproptosis is a highly regulated form of cell death, and its formation and execution are highly governed by the TME [12]. This process is characterized by the immediate interdependence of cellular copper homeostasis and the integrity of the TCA cycle in mitochondria. More to the point, the sensitivity of this axis is ideally positioned to read major hits of the TME, like chronic inflammation and hypoxia [13]. Therefore, understanding every aspect of the molecular machinery of cuproptosis requires researching it within the context of the complex signaling pathways of TME.

### 2.1. Core Pathways of Cuproptosis: From Copper Influx to Mitochondrial Dysfunction

The adverse impact of the accumulation of copper ions within the intracellular space results in cuproptosis [14]. Such a buildup, however, is not incidental in the TME but is a direct outcome of the inflammatory signaling cascade [15], which is characteristic of this site. High levels of pro‐inflammatory cytokines, such as IL‐1β and TNF‐α, act synergistically with the hypoxia‐inducible factor 1‐alpha (HIF‐1α) signaling pathway. Mechanistically, pro‐inflammatory cytokines (TNF‐α and IL‐1β) engage their receptors to trigger the canonical nuclear factor kappa B (NF‐κB) signaling cascade. The activated transcription factors translocate to the nucleus and bind to specific response elements on the SLC31A1 (encoding copper transporter 1, CTR1) promoter, driving its transcriptional upregulation. In the reverse loop, copper overload induces cell rupture and the release of DAMPs, specifically ATP and HMGB1. These ligands bind to purinergic receptors (e.g., P2RX7) and toll‐like receptors (e.g., TLR4) on TAMs. This interaction triggers downstream molecular events, such as NOD‐like receptor family pyrin domain‐containing 3 (NLRP3) inflammasome assembly and further NF‐κB activation, thereby amplifying cytokine production. This creates a molecularly defined positive feedback loop. This, in turn, triggers the fatal chain reaction of tumor cells and makes them highly sensitive to the quantity of copper in the surroundings [16].

**Figure 1:**
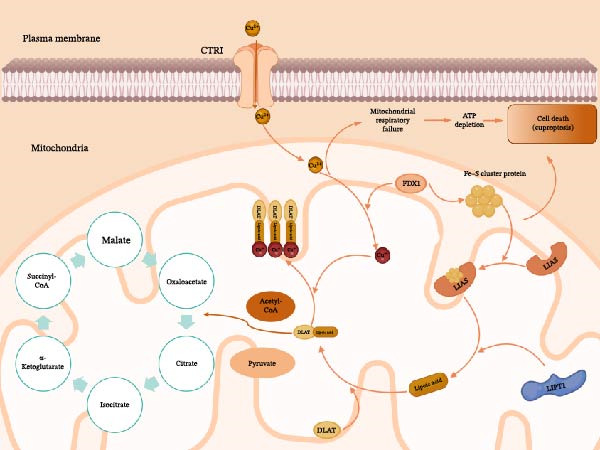
Schematic of the core molecular pathway of cuproptosis excess intracellular copper triggers cuproptosis through a mitochondria‐dependent pathway. Copper ions (Cu^2+^) enter the cell via the CTR1 transporter and are reduced to Cu^+^ by FDX1 in the mitochondria. Cu^+^ then directly binds to lipoylated DLAT, a key protein in the TCA cycle. This binding causes the oligomerization and aggregation of DLAT, which subsequently causes loss of Fe–S cluster proteins. The outcome of the chain reaction culminates in the mitochondrial respiratory failure, the loss of the ATP, and the eventual death of cells. The mechanisms illustrated are summarized from Tsvetkov et al. [11] and Dreishpoon et al. [17]. CTR1, copper transporter 1; FDX1, ferredoxin 1; DLAT, dihydrolipoamide S‐acetyltransferase; TCA, tricarboxylic acid; Fe–S, iron–sulfur. Created with Biorender.com.

The extra copper ions are sent to the mitochondria when they enter the cell. Upon entering the mitochondria, copper initiates a toxic process that is strictly dependent on ferredoxin 1 (FDX1), as illustrated in Figure 1 [18]. FDX1 functions to reduce divalent copper Cu^2+^ into the highly reactive cuprous ion Cu^+^ [11]. This Cu^+^ then directly binds to lipoylated proteins in the TCA cycle, specifically targeting the dihydrolipoamide S‐acetyltransferase (DLAT) subunit of the pyruvate dehydrogenase complex [19]. Recent findings further clarify that FDX1 also regulates the upstream synthesis of these lipoylated targets via the lipoic acid synthetase (LIAS) pathway [17]. The binding of copper causes these proteins to aggregate, leading to proteotoxic stress and the destabilization of Fe–S cluster proteins. These events ultimately destroy mitochondrial respiration and induce cell death [11].

Notably, the vulnerability of a cell to copper‐induced death directly depends on its dependence on mitochondrial respiration and the levels of the FDX1 and protein lipid acylation pathways regulated by genes, including LIAS and lipoyltransferase 1 (LIPT1) [17]. The presence of TME‐inflammatory conditions also has a significant effect on such pathways. It is also evidence of chronic inflammation and hypoxia that strongly regulates tumor cell metabolism [20]. Out of the tumor cells adjusted to the TME through retention of the active oxidative phosphorylation (OXPHOS) and preservation of the TCA cycle, cells are particularly prone to copper‐induced cell death [21]. Conversely, the cells that switch to glycolysis (the so‐called Warburg effect) have an inherent resistance [22]. In this way, the inflammatory properties of the TME regulate the cellular sensitivities to cell death caused by copper using reprograming of metabolism [23]. Simultaneously, oxidative stress caused by chronic inflammation removes glutathione (GSH), the primary intracellular copper chelator, further compromising the cellular defenses and predisposing them to copper toxicity [24, 25].

### 2.2. Distinctions Between Cuproptosis and Other Programed Cell Death Pathways

The therapeutic irreplaceability of cuproptosis stems from its unique molecular target: lipoylated TCA cycle proteins. Unlike ferroptosis, which relies on lipid peroxidation, or pyroptosis, which depends on gasdermin‐mediated membrane pore formation, cuproptosis acts as a direct metabolic checkpoint. It specifically eliminates tumors that depend on mitochondrial respiration (OXPHOS) for survival. Crucially, some aggressive tumors upregulate antioxidant systems to evade ferroptosis but consequently become heavily reliant on mitochondrial metabolism. Cuproptosis exploits this metabolic vulnerability, filling a critical therapeutic gap for tumors resistant to other cell death modalities [26, 27]. The distinct molecular features of cuproptosis compared to other RCD pathways are summarized in Table 1.

**Table 1:** Comparison of cuproptosis with other major RCD pathways.

Characteristic	Apoptosis	Necroptosis	Ferroptosis	Pyroptosis	Cuproptosis
Core trigger	Intrinsic/extrinsic apoptotic signals	Death receptors/PAMPs/DAMPs	Lipid peroxidation	PAMPs/DAMPs	Excess intracellular copper ions
Key execution proteins	Caspase‐3/7/9	RIPK1/RIPK3/MLKL	GPX4 deficiency/ACSL4	Caspase‐1/4/5/11/GSDMD	FDX1/Lipoylated TCA cycle proteins (e.g., DLAT)
Main organelles	Mitochondria/nucleus	Plasmamembrane/mitochondria	Plasmamembrane/mitochondria	Plasma membrane	Mitochondria (TCA cycle)
Morphological characteristics	Cell shrinkage, apoptotic bodies	Cell swelling, plasma membrane rupture	Increased membrane permeability, mitochondrial swelling	Cell swelling, pyroptosome formation	Mitochondrial swelling, protein aggregation
Representative inhibitors	Z‐VAD‐FMK (inhibits Caspase)	Necrostatin‐1 (RIPK1)	Ferrostatin‐1/liproxstatin‐1	VX‐765 (caspase‐1)	Cuproptosis: copper chelators (e.g., tetrathiomolybdate) [substrate depletion]
Immunological consequence	Usually immune suppression	Pro‐inflammatory (ICD)	Pro‐inflammatory (ICD)	High pro‐inflammatory	Pro‐inflammatory (ICD potential)
Key DAMPs	“Don’t eat me” signals	HMGB1 [28], ATP [29], mtDNA [30]	HMGB1 [31], ATP [32]	IL‐1β [33], IL‐18 [34], HMGB1 [35]	CRT, ATP [36], HMGB1 [37], type I IFNs [38]

## 3. Copper Imbalance and Chronic Inflammation in the TME

The cancer cells exist in a complex and dynamic ecosystem that encompasses a variety of nonmalignant cells, such as immune cells, fibroblasts, and endothelial cells, found within the context of the extracellular environment (TME) [39]. The two important attributes of the TME are chronic inflammation and metabolic reprograming, which are often related closely and play an important role in tumor growth and adaptive response to therapy [40]. This interaction, according to recent studies, is dependent on copper metabolism. It contributes to carcinogenesis and targeted therapeutic activity.

### 3.1. The Phenomenon of “Copper Addiction” in Cancer

Zhou et al. [41] analyzed pan‐cancer data and found aberrant cuproptosis regulators. Ling et al. [42] identified high copper levels in lung adenocarcinoma. Furthermore, Liao et al. [43] revealed that inflammation drives copper accumulation in colorectal cancer via the STEAP4 axis. Researchers have referred to this accumulation as an active habit, not just a passive process. Copper is necessary in several essential functions performed by cancer cells, which causes the development of tumors.

Copper is highly necessary in angiogenesis, which is the creation of new blood vessels that the tumors need to grow and obtain nutrients [44]. Copper interacts with proangiogenic factors and networks; it activates endothelial cell migration and proliferation in association with augmented production and secretion of vascular endothelial growth factor (VEGF) and increases proangiogenic networks [45, 46]. Copper, in addition to causing angiogenesis, also directly interferes with intracellular signaling pathways to sustain cell survival and proliferation. The copper is needed to activate the BRAF‐MEK‐ERK mitogen‐activated protein kinase (MAPK) pathway, which is known to cause cancer and is a key signaling pathway. Copper is a direct agonist of MEK1/2 kinases that interacts with them to enhance the activity of the latter, and thus, stimulate the proliferation signal necessary to trigger the tumor proliferation [47].

The adoption of copper addiction and the new studies have made recent discoveries that have shed more light on the molecular components of addiction. In bioinformatics designing and testing, a positive correlation has been reported with the same expression of epithelial–mesenchymal transition‐related genes in high copper levels through the entire range of colorectal cancer. This fact indicates that disturbed copper metabolism is a decisive element of the mechanisms of tumor metastasis [48]. All these results prove that copper dependence is one of the primary factors that causes the development of malignant tumors.

### 3.2. Inflammatory Regulation of Copper Homeostasis in the TME

One of the characteristics of cancer is chronic inflammation. The chronic inflammatory condition within the TME contributes to tumor formation, progression, and metastasis in various ways [49]. A complicated and bidirectional interdependence exists between this inflammatory condition and copper homeostasis. Pro‐inflammatory cytokines that are quite prolific in the TME can dynamically control the expression and activity of the cell‐surface copper transporter proteins.

For example, mechanisms observed in other chronic inflammatory conditions (e.g., Parkinson’s disease) demonstrate that TNF‐α and IL‐1β can upregulate CTR1 while downregulating the copper exporter ATP7A [50]. Given the similar inflammatory milieu in the TME, we speculate that a comparable cytokine‐driven axis may also promote copper retention in tumor cells. Pro‐inflammatory signals enhance CTR1 expression, thus leading to the increased copper uptake and retention in tumor cells and immune cells (e.g., macrophages) in the TME in most cases. Such a localized increase in copper can also encourage copper‐addiction‐related activities of tumor cells. It can also, on the other hand, create conditions that are favorable to the toxicity caused by copper (cuproptosis), hence offering a possible therapeutic window.

The other characteristic of the TME is hypoxia, which makes the situation even more complex since the clinical axis of tumor development is regulated [51]. HIF‐1α, which plays an important role in the central regulation of cellular hypoxic response, is highly synergized with robust inflammatory signaling, especially NF‐κB. All these pathways modify cellular metabolism and angiogenic pathways. It is important, as HIF‐1α directly controls several copper metabolism and lipid acylation‐related genes, including the *SLC31A1 (CTR1)* and the *LIAS* ones [16]. It is a physiologically important control since it guarantees that copper‐dependent enzymes, such as cytochrome c oxidase, which contributes to mitochondrial respiration, are provided with adequate copper to only allow mild oxidative phosphorylation without enough oxygen.

**Figure 2:**
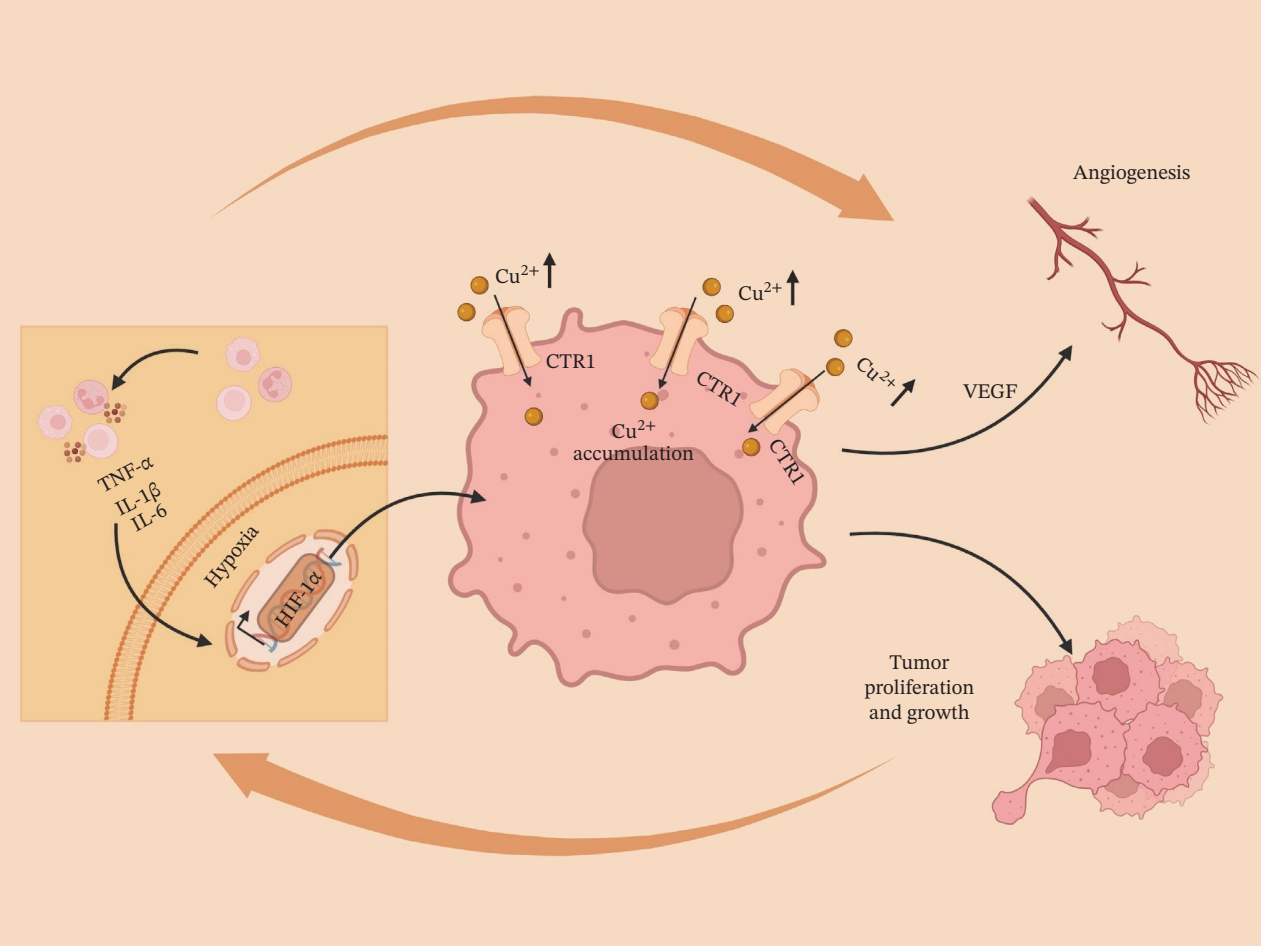
Copper addiction in the tumor microenvironment and its vicious cycle with inflammation. Immune cells produce hypoxia (through the HIF‐1α) and pro‐inflammatory cytokines (IL‐1β and TNF‐α). These are combined to induce tumor cells to express the copper transporter CTR1 more. The buildup of intracellular copper (Cu^2+^) encourages angiogenesis, cell growth, and tumor expansion. The process forms a positive feedback mechanism that maintains and reinforces the malignancy of the cancer cells. The regulation of CTR1 by inflammatory cytokines and hypoxia is summarized from recent studies [16, 50]. Created with Biorender.com.

Hypoxia and chronic inflammation drive metabolic reprograming within the TME. The demand for copper is the highest in this environment, with increased consumption of copper and changes in copper transportation. Instead, these alterations lead to a phenotype that does not only help the cultivation of tumors but also determines the metabolic status of the cells, which become insensitive to cuproptosis. This dysregulation is a part of the complex and conflicting system of control. The reciprocal relationship between inflammatory signaling and copper accumulation in tumor cells is illustrated in Figure 2.

### 3.3. Adaptive Resistance: Evading Cuproptosis

Tumor cells develop sophisticated adaptive strategies to evade copper toxicity while maintaining essential metabolic functions. The most direct mechanism involves regulating copper transport kinetics. Cells limit copper accumulation by downregulating the influx transporter CTR1 or upregulating efflux pumps, such as ATP7A and ATP7B [52]. Beyond these transport adjustments, tumors employ complex metabolic rewiring as a deeper form of adaptive resistance. Since cuproptosis specifically targets lipoylated proteins within the TCA cycle, cancer cells can adaptively shift their metabolic dependency from oxidative phosphorylation to glycolysis (Warburg effect). This metabolic plasticity allows them to bypass the mitochondrial vulnerabilities targeted by copper. Thamarai Kannan et al. [53] demonstrated that SHLP6 upregulates GSH and antioxidant enzymes to mitigate copper‐induced toxicity. Additionally, Zhao et al. [54] observed that low FDX1 expression correlates with poor prognosis, suggesting tumors downregulate FDX1 to raise the threshold for cell death. Understanding this multilayered resistance—spanning transport regulation, metabolic reprograming, and stress response—is crucial for designing effective combination therapies.

## 4. Bidirectional Interactions Between Cuproptosis and TME Inflammation

**Figure 3:**
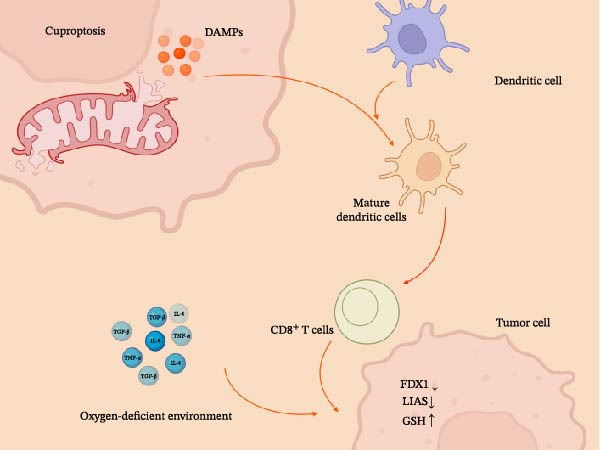
Bidirectional regulatory network between cuproptosis and the inflammatory state of the tumor microenvironment. Cuproptosis is an immunogenic cell death resulting in the release of DAMPs that stimulate the maturation of DCs and the consequent activation of cytotoxic CD^8+^ T lymphocytes. This transforms the tumor into a “hot” state rather than a “cold” state. Alternatively, the immunosuppressive TME has the ability to alter the concentrations of key mediators such as FDX1, LIAS, and GSH to predispose or anti‐dispose tumor cells to experience cuproptosis. It is facilitated by hypoxia and signaling factors such as TNF‐α, IL‐6, and TGF‐β. This mutually interactive process contributes to tumor eradication by the immune system or facilitates immune evasion and resistance to treatment. The immunogenic signaling pathways and DAMPs release are depicted based on preclinical data from Luo et al. [55] and Wu et al. [56]. Created with Biorender.com.

Even though one can now know more about the links between cuproptosis and copper homeostatic balance in the TME, one of the most significant gaps is still the other way around: the dynamic, two‐way relationship between cuproptosis and chronic inflammation. This occurs unidirectionally, as the mechanism of cuproptosis can influence the associated inflammatory milieu, and conversely, the existence of preexisting inflammation may vary the threshold for initiating cuproptosis. This section discusses this complicated connection and introduces a novel idea: cuproptosis could be a key link between metal metabolism and the immune system’s ability to fight cancer. The bidirectional regulatory network between cuproptosis and TME inflammation is depicted in Figure 3.

### 4.1. Remodeling the TME Immune Landscape via Cuproptosis

It is now known that the effectiveness of many cancer treatments, such as chemotherapy and radiotherapy, is partly based on their ability to kill cells, which leads to an immune response against tumors. This observation prompts a vital inquiry: Is cuproptosis immunogenic?

#### 4.1.1. Immunogenic Potential of Cuproptosis and DAMPs Profiling

ICD is a unique type of programed cell death whose main defining feature is the ability to allow dying cells to spatially and temporally expose or release a spectrum of DAMPs [57]. These can also serve as potent endogenous immune adjuvants, activating and directing the adaptive immune system to attack the tumor [58]. ICD presents with the hallmark proximal triad, which includes (1) calreticulin (CRT) exposure on the cell surface, (2) active ATP secretion, and (3) passive release of HMGB1 [59].

Because cuproptosis is an oxidative process triggered by severe proteotoxic stress and dysfunction in the mitochondria, it is also probable to be commonly characterized by some main characteristics with ICD:

CRT exposure: copper‐induced aggregation of lipoylated proteins induces severe endoplasmic reticulum stress and unfolded protein response (UPR), another downstream event that results in the translocation of CRT to the cell surface. CRT displayed on the surface is identified by receptors on the phagocytes, especially DCs, which substantially increases the phagocytosis of the dying tumor cells [60].

ATP release: the central location of intracellular ATP generation is the mitochondria. Membrane loss and respiratory chain collapse in the mitochondrion are observed during cuproptosis. Consequently, large quantities of ATP are released through mitochondria into the cytoplasm and externalized through pore‐like channels like pannexin‐1. Extracellular ATP can be an essential stimulus acting on P2RX7 receptors of DCs and macrophages to facilitate inflammasome assembly and release of IL‐1β [61].

Release of HMGB1: HMGB1 ordinarily resides in the nucleus and is passively released when cell membrane integrity is compromised. Upon its release, it attaches to pattern recognition receptors (e.g., TLR4 and RAGE) on DCs and macrophages to induce maturation of these cells and release pro‐inflammatory cytokines.

Potential cyclic GMP‐AMP synthase‐stimulator of IFN genes (*cGAS-STING*) activation: severe mitochondrial stress can trigger the cGAS‐STING pathway through the leakage of mitochondrial DNA (mtDNA), as seen in other contexts [62]. Since cuproptosis involves mitochondrial protein aggregation and respiratory collapse, it might theoretically induce similar signaling. Importantly, Zhu et al. [38] recently demonstrated that cuproptosis triggers mitochondrial proteotoxic stress, leading to the release of mtDNA into the cytoplasm. This event activates the cGAS‐STING signaling pathway, subsequently promoting the secretion of type I IFNs (IFN‐β) and facilitating DC maturation. This provides direct evidence linking cuproptosis to innate immune activation. The synergistic interaction of several DAMPs achieves strong immunology. The combination of these signals serves well to recruit and wake up the antigen‐presenting cells within the TME, namely the cDC1 subgroup. Activated DCs absorb tumor cells into them, digest them, and process tumor antigens to the surface in the form of major histocompatibility complex class I (MHC‐I) molecules. These DCs then move to the draining lymph nodes and activate tumor‐specific CD8^+^ T cells [63]. This results in the induction of a strong polyclonal tumor‐specific cytotoxic T‐lymphocyte (CTL). Subsequently, noninflammatory tumors, which are immunologically cold and devoid of infiltration by T‐cells, could be turned into hot tumors with high CTL activation. Such transformation is essential to improve the effectiveness of immune checkpoint inhibitors (ICIs) [64].

Recent preclinical studies provide initial evidence linking cuproptosis to immunogenic signals. Luo et al. [55] developed a copper and elesclomol codelivery system. They observed that inducing cuproptosis led to the release of ATP and HMGB1 in breast cancer cells. In coculture experiments, these dying cells increased CD80 and CD86 expression on DCs. Similarly, Wu et al. [56] reported that copper‐based treatment increased CRT exposure on the cell surface. This was associated with higher CD8^+^ T cell infiltration in their model. These findings suggest that cuproptosis might share features with ICD and could help alter the immune environment of “cold” tumors, though more direct evidence is needed to fully define its immunogenic profile.

Beyond standard DAMPs, cuproptosis involves severe proteotoxic stress and protein aggregation. It remains unclear if these copper‐induced protein aggregates interact with the immune system. Misfolded proteins from other cell death pathways can bind to scavenger receptors on phagocytes and trigger inflammation [65]. Therefore, we speculate that cuproptotic protein aggregates might serve as unique danger signals or inflammatory triggers. However, strictly speaking, whether these specific aggregates possess immunogenicity requires further experimental validation.

#### 4.1.2. Differential Sensitivity of Immune Cell Subpopulations to Copper‐Induced Cell Death

The effects of copper work not only on tumor cells but also on the different immune cells. The subpopulations of immune cells in the TME exhibit different metabolic phenotypes that lead to differences in the sensitivity of specific immune cells to copper‐induced cell death. In addition to the above‐described immunosuppressive cells, it is important to consider the larger immunological situation. Immunosuppressive regulatory T cells (Tregs) and M2‐type TAMs rely heavily on mitochondrial OXPHOS for survival [66, 67]. Since cuproptosis targets the TCA cycle, we theorize that these cells might be more sensitive to copper toxicity [68]. In contrast, effector T cells (Teff) and natural killer (NK) cells mainly use aerobic glycolysis [69, 70]. This metabolic feature could theoretically make them less sensitive. However, these are predictions based on metabolic patterns (Table 2). We lack direct evidence linking these metabolic traits to cuproptosis sensitivity.

**Table 2:** Theoretical prediction of immune cell sensitivity based on metabolic phenotypes.

Immune cell subtype	Core metabolic feature	Key copper proteins (expression)	Predicted cuproptosis sensitivity
Effector T cell (Teff)	Aerobic glycolysis	Moderate CTR1, low ATP7A/B	Low to moderate
Regulatory T cell (Treg)	Oxidative phosphorylation (OXPHOS)	High CTR1, moderate ATP7A/B	High [71]
M2‐TAM	OXPHOS, fatty acid oxidation	High CTR1, low ATP7A	High
M1‐TAM	Aerobic glycolysis	High CTR1, high ATP7A	Moderate
Natural killer (NK) cell	Aerobic glycolysis	Moderate CTR1	Low to moderate
Dendritic cell (DCs)	Metabolically flexible (glycolysis/OXPHOS)	Variable	Context‐dependent
B cell (activated)	Aerobic glycolysis	Moderate CTR1	Low to moderate

On the other hand, such cells characterized by metabolic plasticity, such as DCs and B cells that can dynamically oscillate between oxidative phosphorylation and glycolysis as a response to activation, would tend to be context sensitive. If confirmed, this metabolic difference could offer a therapeutic window. Copper agents might selectively deplete immunosuppressive cells. This could help lower the immune barrier in the TME. However, current research has not fully explored this selective killing in vivo. It is also unclear if this mechanism can truly reshape the immune microenvironment to benefit patients. Future studies must verify if copper‐based therapies can achieve this precise modulation in clinical settings. Based on their distinct metabolic dependencies, we hypothesize that immune cell subsets may exhibit differential sensitivity to cuproptosis, as outlined in Table 2.

### 4.2. Regulation of the Threshold for Copper‐Induced Cell Death by the Inflammatory Microenvironment

The inflammatory disease of the TME actively controls cell adhesion to cell death, in addition to arising in response to the cell death caused by copper. This dual control is achieved by direct cytokine action and indirect action through metabolic reprograming.

#### 4.2.1. Direct Regulation by Cytokine Signaling

Cytokine inflammatory mediators have the potential to directly mediate the molecular processes of inflammatory cell death that copper induces. For example, TNF‐α, a prominent pro‐inflammatory cytokine in most TMEs, mainly interacts with the NF‐κB pathway [72]. NF‐κB activation can potentially control the transcription of important genes implicated in copper‐induced cell death. Thus, TNF‐α can increase FDX1, a master mediator of copper‐induced cell death in highly inflamed conditions, and so increase the sensitivity of the cancer cells to copper toxicity. Conversely, anti‐inflammatory/pro‐fibrotic cytokines such as transformed growth factor‐β (TGF‐β) can enhance cellular antioxidant responses and regulate mitochondrial activity. As an illustration, TGF‐β can increase the GSH or cause a change in the mitochondrial bioenergetics, thereby increasing the threshold of copper‐induced cell death and inducing drug resistance.

#### 4.2.2. Indirect Regulation via Inflammation‐Induced Metabolic Shifts

In addition to direct signaling, chronic inflammation is a potent initiator of metabolic reprograming in TME. Such an inflammatory state can cause inflammatory and immunological cells to persist in more dependence on glycolysis, which is an exclusive metabolic event called the Warburg effect [73]. This decreases the dependence of the TCA cycles, leading to an increased resistance toward copper‐induced cytotoxicity, as suggested earlier. These reactions form a vicious cycle: long‐term inflammation raises copper concentration in the region yet also triggers the development of altered cellular metabolism such that they do not die of excessive copper load. We should value this metabolic escape pathway to make effective copper anticancer treatments. These observations also indicate that a cotargeting approach of the glycolysis and cuproptosis pathways may resensitize tumors to copper toxicity in an inflammatory state. In conclusion, copper‐induced cell death acts as a bottleneck at which inflammation and metabolic rewiring mutually determine cell fate in the TME. Furthermore, lipid metabolism plays a crucial role in determining cell fate during copper stress. Since cuproptosis triggers cell death by targeting lipoylated TCA cycle proteins, alterations in fatty acid oxidation and lipid synthesis pathways can significantly modulate tumor cell sensitivity to copper‐induced toxicity.

## 5. A New Therapeutic Paradigm for Reshaping the Tumor Inflammatory Microenvironment

Considering the dramatic and bidirectional interactions of copper‐induced cell death and the TME, therapeutic interventions are shifting beyond simply causing tumor cell apoptosis. Regarding recent innovations, Xu et al. [74] engineered triphenylphosphine‐chitosan functionalized MoS2 nanosheets for targeted copper delivery to mitochondria to inhibit tumor growth. Additionally, Zhang et al. [75] designed a ZnO@CuEA nanoplatform. They showed that this platform released copper ions in the acidic TME, achieving synergistic therapy through cuproptosis. Instead, these approaches can be viewed as a different paradigm, namely, the remodeling of the immune environment of the TME and the inversion of immunosuppression. The main idea of such an approach is to mechanically transform the immunologically changed cold immunosuppressive TME, where immune cell penetration does not occur, into the hot TME full of inflammatory cues and as immune cell‐permissible to take place. Such a shift will offer the chance to improve the current immunotherapies, including ICIs, to achieve optimal synergistic effects.

### 5.1. Inducing Immunogenic Copper‐Induced Cell Death to Activate “Cold” Tumors

Its main approach to the therapeutic use of copper‐induced cell death is to pharmacologically trigger this process, thereby inducing ICD to reactivate the immune microenvironment of “cold” tumors [76]. In contrast to the comparatively mild form of cell death, apoptosis, copper‐sensitive cell demise is associated with disastrous protein folding and mitochondrial malfunction [77]. This radical homeostatic perturbation accelerates the chances of the release or exposure of DAMPs to a high degree [78]. DAMPs, such as ATP (find me) and HMGB1 (danger)—signals that have been usefully recognized as pertinent to DCs—play an essential role in stimulating adaptive antitumor immunity. Inflammatory TME is exceptionally responsive to pharmacological agents like copper ion carriers (e.g., elesclomol) [79]. In this case, the upregulation of the copper transporter CTR1 (activated by chronic inflammation and hypoxia) and a decreased intracellular GSH concentration (hypoxia‐induced oxidative stress) provide an opportunity period in which copper will induce cell death quickly and efficiently [80]. Increased copper cytotoxicity increases tumor neoantigen exposure and intubation of immunostimulatory signals [81]. The effect of this procedure is on “cold” tumors that, initially, are nonresponsive and makes them responsive to ICIs like anti‐PD‐1/PD‐L1 antibodies [82]. The ultimate outcome is alleviating T cell exhaustion and attaining synergistic antitumor effects, as observed by Peng et al. [83] in colorectal cancer models treated with CuS/MnO_2_/diAMP nanoparticles.

**Figure 4:**
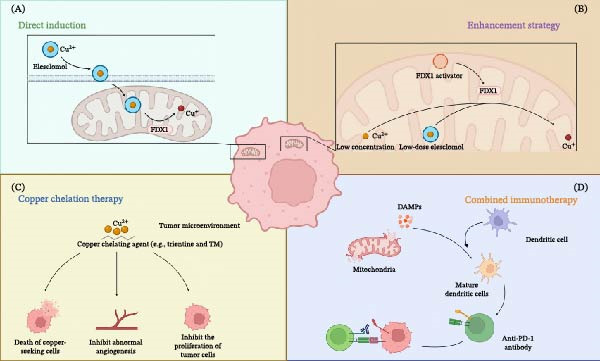
Therapeutic strategies targeting copper metabolism for cancer treatment. (A) Direct induction of cuproptosis. Copper ionophores, such as elesclomol, facilitate the efficient transport of copper ions into cancer cells that are highly dependent on mitochondrial respiration. The subsequent intracellular accumulation of copper in the mitochondria triggers FDX1‐mediated protein lipoylation and aggregation, ultimately inducing cuproptosis. (B) Sensitization strategy for cuproptosis. Small‐molecule drugs, such as FDX1 activators, can lower the threshold for cuproptosis. This sensitizes cancer cells, rendering them susceptible to cell death even at physiological copper concentrations or with low, subtoxic doses of copper ionophores. (C) Copper chelation therapy. Copper chelators, such as trientine, deplete copper ions within TME. This strategy aims to “starve” copper‐addicted tumors by inhibiting key copper‐dependent processes, including abnormal angiogenesis and tumor cell proliferation, and may selectively kill copper‐seeking cells. (D) Combination with immunotherapy. The induction of cuproptosis promotes ICD, characterized by the release of DAMPs and tumor‐associated antigens. This process facilitates DCs maturation and subsequent antigen presentation to T cells. The combination with ICIs, such as anti‐PD‐1 antibodies, synergistically enhances the anti‐tumor immune response by reinvigorating exhausted T cells. Strategies illustrated include copper ionophores [11], combination immunotherapies [84], and chelators [86, 87]. Created with Biorender.com.

The synergistic approach has proven to be effective in several preclinical models. Recent advancements in nanomedicine provide concrete examples of this synergy. Lu et al. [84] developed an acid‐responsive elesclomol‐loaded copper oxide nanoplatform (ES@CuO). Their study on B16F10 melanoma models demonstrated that ES@CuO treatment not only triggered cuproptosis but also induced robust reactive oxygen species (ROS) generation, leading to the release of DAMPs. Crucially, when combined with anti‐PD‐1 therapy, this strategy significantly increased the infiltration of CD8^+^ T cells while reducing the populations of immunosuppressive MDSCs and Tregs, successfully sensitizing the tumors to checkpoint blockade. Mechanistically, Zheng et al. [85] found that mitochondria‐targeted photothermal therapy combined with checkpoint blockade increased CD8^+^ T cell infiltration and IFN‐γ activity, effectively turning “cold” tumors into “hot” tumors. These encouraging findings provide a good case to justify the clinical testing of copper‐induced cell death inducers in complex with ICIs. The major therapeutic strategies targeting copper metabolism for cancer treatment are summarized in Figure 4.

### 5.2. Modulating TME Inflammation With Copper Chelators

Unlike direct induction of cell death, copper chelators provide a better‐purified method of controlling inflammation in the TME [88–90]. Instead of direct tumor cell lysis, copper chelation decreases copper ion bioavailability and interferes with the metabolic basis of chronic inflammation, angiogenesis, and immunosuppression in the TME [91–93]. Copper ions are some of the most important cofactors of various enzymes, and many protumorigenic processes in the TME depend on this type. Notably, Cox et al. [94] demonstrated that tetrathiomolybdate (TM) inhibits lysyl oxidase (LOX) in breast cancer models, preventing collagen cross‐linking and reducing stiffness. This LOX‐inhibitory effect was further corroborated by subsequent studies [95]. Zeng et al. also combined hepatic artery ligation with copper chelators [86], confirming that this strategy effectively suppresses tumor angiogenesis and HIF‐1α expression. Consistently, studies indicate that immunosuppressive cells, such as MDSCs and Tregs, require sufficient copper to promote their proliferation and functional responses within the metabolically stressed TME [96, 97]. Thus, the potentially harmful effects of copper chelation therapy might be used to selectively destroy the survival and functionality of such cells, eliminating immunosuppression. Consequently, copper chelation is a versatile agent, an anti‐angiogenic agent, and a TME normalizer. This methodology helps correct metabolic impairments throughout the inflammatory microenvironment and, in doing so, establishes more conducive environments for other immunotherapies.

Copper chelation therapy has already advanced to the stage of clinical evaluation. An illustration is that phase II clinical trials of another copper chelator, TM, have been conducted on patients with advanced solid tumors, like breast cancer. In a clinical study, Liu et al. [87] found that TM treatment reduced MDSCs and Tregs in high‐risk breast cancer patients, while being generally well tolerated. Other copper chelators, of which trientine was the first drug to be given the green light in the treatment of Wilson’s disease, are currently being evaluated in cancer therapy, particularly during chemotherapy or immunotherapy. Fu et al. [98] reported that a copper‐lowering agent overcame platinum resistance in preclinical models. Subsequently, they conducted an exploratory clinical study combining carboplatin with trientine in patients with advanced malignancies [99], justifying further clinical investigation.

### 5.3. Precise Immune‐Metabolic Coregulation via Smart Nanomedicine

**Figure 5:**
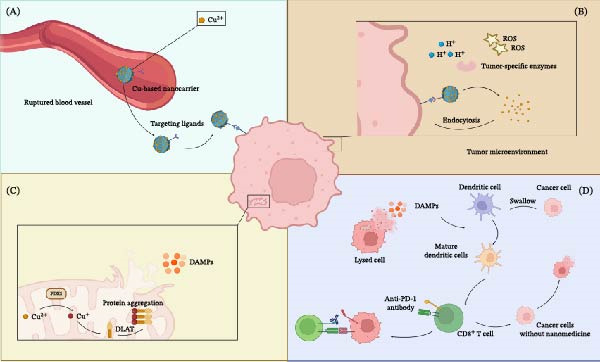
Smart nanomedicine strategies for inducing immunogenic cuproptosis and remodeling the tumor microenvironment. (A) Targeted delivery and accumulation of copper‐based nanocarriers. Copper‐based nanocarriers, functionalized with targeting ligands, are administered systemically. They leverage the enhanced permeability and retention (EPR) effect to extravasate through ruptured tumor blood vessels and accumulate in the tumor microenvironment. The targeting ligands then facilitate specific binding and uptake by cancer cells. (B). TME‐responsive release of copper ions. When cancer cells take in the nanocarriers by endocytosis, they are exposed to the TME, which has its own set of circumstances, such as low pH (H^+^), high amounts of ROS, and/or enzymes that are peculiar to tumors. These triggers cause the nanocarrier to break down, which releases copper ions (Cu^2+^) inside the cell. (C) Intracellular induction of cuproptosis and immunogenic cell death. The copper ions that are released build up in the mitochondria. In this case, FDX1 helps Cu^2+^ change into the more hazardous Cu^+^, which subsequently binds to lipoylated proteins like DLAT. This binding causes these proteins to stick together, which leads to proteotoxic stress, problems with the mitochondria, and eventually cell death through cuproptosis. This mechanism triggers an immune response, which leads to the release of damage‐associated molecular patterns (DAMPs). (D) Remodeling the TME and combination with immunotherapy. The discharged DAMPs are used as “find‐me” signals, which promote the formation and recruitment of DCs. The DCs consume the dead cancer cells, digest the tumor‐related antigens, and present them to CD^8+^ T lymphocytes. This initiates a robust anti‐tumor immune response that may also target other cancer cells. Nanomedicine can be used to enhance this immunological cascade further by using it with immune checkpoint inhibitors, such as anti‐PD‐1 antibodies. These antibodies prevent the process of fatigue in T cells and amplify the anti‐tumor effect of the system as a whole. This schematic illustrates the design principles of TME‐responsive nanoplatforms as reported by Lu et al. [84] and Wu et al. [56]. Created with Biorender.com.

Innovative nanotechnology can provide spatiotemporal control in order to balance the physiological activity and toxicity of copper ions, as well as to maximize copper‐targeted therapies. These nanoparticles can respond to tumor tissue‐specific inflammatory characteristics such as acidic pH, high levels of ROS, or high activity of matrix metalloproteinases, allowing release of the selective payload upon reaching the tumor tissue [100–102]. The toxicity of copper‐based treatments and their narrow therapeutic index are especially detrimental to developing copper‐based therapies, as it provides a serious impediment to clinical translation, especially in children who are highly sensitive to copper in organs such as the liver, kidneys, and nervous system. To avoid these challenges, intelligent nanocarriers are being developed to attain tumor‐specific delivery. The latter is affected by passive accumulation via the enhanced permeability and retention (EPR) effect and, more efficiently, active targeting, that is, by cancer cell receptors. Tumors tend to overexpress receptors recognized with ligands (e.g., antibodies, peptides, and aptamers) on the nanoparticle’s surface [103]. To provide additional accuracy, the systems may be stimulus–responsive to release their payload, either through an intrinsic response to an acidic environment or by releasing tumor‐specific proteases. This dual‐targeting approach will vastly increase the therapeutic index by targeting and focusing on dual escalation of the intratumoral drug concentration and simultaneously reducing off‐target toxicity. Also, the nanocarriers permit an intricate immuno‐metabolic synergistic therapy by delivering various functional agents. Rationally compounded nanoparticles can codeliver a copper complex to cause ICD and an immunomodulator, such as a STING agonist or ICI, to enhance immune responses in an inflamed microenvironment [104, 105]. When targeted successfully and on‐demand cargo release occurs, copper‐induced cell death may initially trigger immune responses in otherwise cold tumors, thereby establishing a critical period of immune activation. This window can then be fully utilized by the timely release of immunomodulators, which generate the accurate institutions of time and space synchronization in the induction of inflammation and immune potentiation. This approach embodies one of the general trends in the direction of more efficient and more specific tumor immunotherapy. The design and mechanism of smart nanomedicine for inducing immunogenic cuproptosis are illustrated in Figure 5.

### 5.4. Challenges in Clinical Translation and Future Prospects

Despite the theoretically vast potential for copper‐induced cell death to impact TME inflammation, transforming preclinical research ideas into everyday clinical practice remains quite challenging.

A critical bottleneck lies in the specific safety profiles of different agents. Copper ionophores (e.g., elesclomol) face the risk of systemic toxicity due to off‐target accumulation, potentially causing severe damage to copper‐rich organs like the liver, kidneys, and nervous system. Conversely, copper chelators present a risk of “accidental injury” to normal physiology. Systemic copper depletion can inhibit essential enzymes, such as LOX [94, 95] and cytochrome c oxidase [16], leading to potential metabolic or structural dysfunction. Thus, achieving a therapeutic window that kills tumors without impairing essential physiological copper functions remains difficult.

While nanomedicines offer targeted delivery, their clinical translation is hindered by biological barriers. The efficiency of passive targeting relies heavily on the EPR effect, which is often heterogeneous or absent in human solid tumors compared to animal models. This variability limits the universal penetration of nanodrugs. Furthermore, safety concerns extend to combination immunotherapies. The massive release of DAMPs by cuproptosis, when combined with checkpoint inhibitors, carries the potential risk of immune overactivation, specifically cytokine release syndrome. Evaluating the balance between anti‐tumor immunity and systemic immune toxicity is therefore essential.

As the discussion in Section 3.3 points out, cancer cells can also acquire resistance by turning down FDX1, turning up copper efflux pumps, or increasing antioxidant defenses. Then, this resistance to combination therapies needs careful research to have strategies for overcoming or preventing this resistance.

Further studies are recommended to systematically agglomerate the profile of DAMPs generated during cuproptosis and map the downstream immune pathways that follow cuproptosis. The production of new and selective cuproptosis modulators, which are safer, is also central. It is also necessary to systematically investigate their synergy with other treatments (e.g., chimeric antigen receptor T cells, cancer vaccines, and radiotherapy). Such investigations should aim to validate whether cuproptosis can function as an in situ vaccine to potentiate these combination therapies.

## 6. Future Perspectives

### 6.1. Key Scientific Questions to be Addressed

Despite the significant progress, copper‐induced cell death research is still in its early stages. Some fundamental scientific questions are open to answers, and their answers will define the field’s future direction and clinical implementation. First, more sophisticated experimental models are urgently required than usual in vitro models to clarify the role of copper‐induced cell death in tumor initiation, progression, metastasis, and treatment response. Second, the impact of the complex physicochemical TME on cuproptosis efficiency remains unclear. Clinical tumors are characterized by specific conditions such as acidic pH, high ROS, and nutritional deficiencies. These harsh environmental stressors could dynamically regulate copper bioavailability or the stability of lipoylated proteins. Current studies mostly use standard culture conditions that fail to mimic these physiological realities. Therefore, future work must determine whether these factors promote or inhibit copper‐induced cell death to predict clinical efficacy accurately. In addition, although hypothetically promising, the immunogenic nature of copper‐induced cell death is still mostly hypothetical and needs to be proved by solid in vivo and ex vivo experiments. Last, highly specific pharmacological modulators with minimal off‐target effects are lacking; as such, new drugs that would target key elements of this pathway are essential to ensure safety and successful application in clinical practice.

### 6.2. Future Research Directions and Prospects

To continue, a multidisciplinary approach and new methodological developments must be implemented to address these challenges related to copper‐induced cell death. Using high‐tech technologies such as single‐cell multiomics and spatial transcriptomics, researchers will investigate the systematic analysis of cell‐type‐specific sensitivity, and the precise mapping of time–space changes in copper‐mediated cell death in the TME. Genetic engineering of highly sophisticated mouse models and manipulation is expected to play a defining role in non‐in vitro clarification of physiological roles in vivo. In addition to that, novel nanomedicine delivery models enable selective delivery of copper modulators to tumors to improve therapeutic effects and minimize toxicity. Remarkably, the biology of copper‐induced cell death may have far‐reaching implications beyond oncology, and additional research should be conducted to include the application of copper‐induced cell death in neurodegenerative diseases, cardiovascular diseases, and other diseases related to copper dysregulation and chronic inflammation. These fundamental, translational, and clinical research questions are the most effective to be systematically answered, enabling the therapeutic potential of copper‐induced cell death to be achieved, opening the door to novel therapies that can provide significant patient advantages.

## 7. Conclusion

Through this review, we have outlined why cell death caused by copper is important in connecting cellular metabolism to the inflammatory status of the TME. This would help better define the bidirectional processes involved and strengthen our knowledge of remodeling antitumor immunology toward creating the next generation of cancer therapy that acts on copper metabolism. It will play an important role in defining upcoming approaches to cancer treatment and patient outcomes.

## Funding

This work was supported by the Shandong Provincial Natural Science Foundation (Grant ZR2025QC927), the Medical and Health Science and Technology Project of Shandong Province (Grant 202502050518), the Binzhou Hospital of Traditional Chinese Medicine Doctoral Research Fund (Grant ZYY2024BSKY02), and the Special Project for Traditional Chinese Medicine Technological Innovation Project of Binzhou Medical University (Grants 2024ZYYZX06 and 2024ZYYZX08).

## Conflicts of Interest

The authors declare no conflicts of interest.

## Data Availability

Data sharing is not applicable to this article as no datasets were generated or analyzed during the current study.
